# Supplementary material for: Sequence controlled secondary structure is important for the site-selectivity of lanthipeptide cyclization
Source: Chem Sci. 2023 May 9;14(25):6904–14. doi: 10.1039/d2sc06546k (PMC10306099; doi:10.1039/d2sc06546k)
Supplement: SC-014-D2SC06546K-s001 [file SC-014-D2SC06546K-s001.pdf]

Electronic Supplementary Material (ESI) for Chemical Science.
This journal is © The Royal Society of Chemistry 2023

# Electronic Supplementary Information: Sequence Controlled Secondary Structure Is Important for the Site-selectivity of Lanthipeptide Cyclization

Xuenan Mi,[a] Emily K. Desormeaux,[b] Tung T. Le,[b] Wilfred A. van der Donk, *[b] and Diwakar Shukla *[acd]

[a]Center for Biophysics and Quantitative Biology, University of Illinois at Urbana-Champaign, Urbana, IL, 61801
[b]Department of Chemistry and Howard Hughes Medical Institute, University of Illinois Urbana-Champaign, Urbana, IL, 61801. Email: vddonk@illinois.edu
[c]Department of Chemical and Biomolecular Engineering, University of Illinois at Urbana-Champaign, Urbana, IL, 61801
[d]Department of Bioengineering, University of Illinois Urbana-Champaign, Urbana, IL 61801. Email: diwakar@illinois.edu

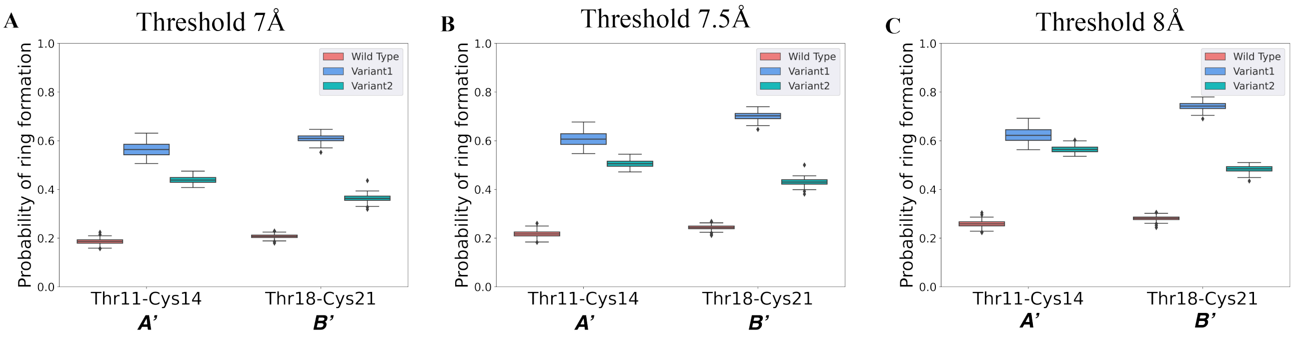


Figure S1: Probability of formation of ring A$'$ (Thr11/Cys14) and B$'$ (Thr18/Cys21) for ProcA3.3 variants using different thresholds. Error bar was calculated by 200 bootstrap samples with 80% of total trajectories.

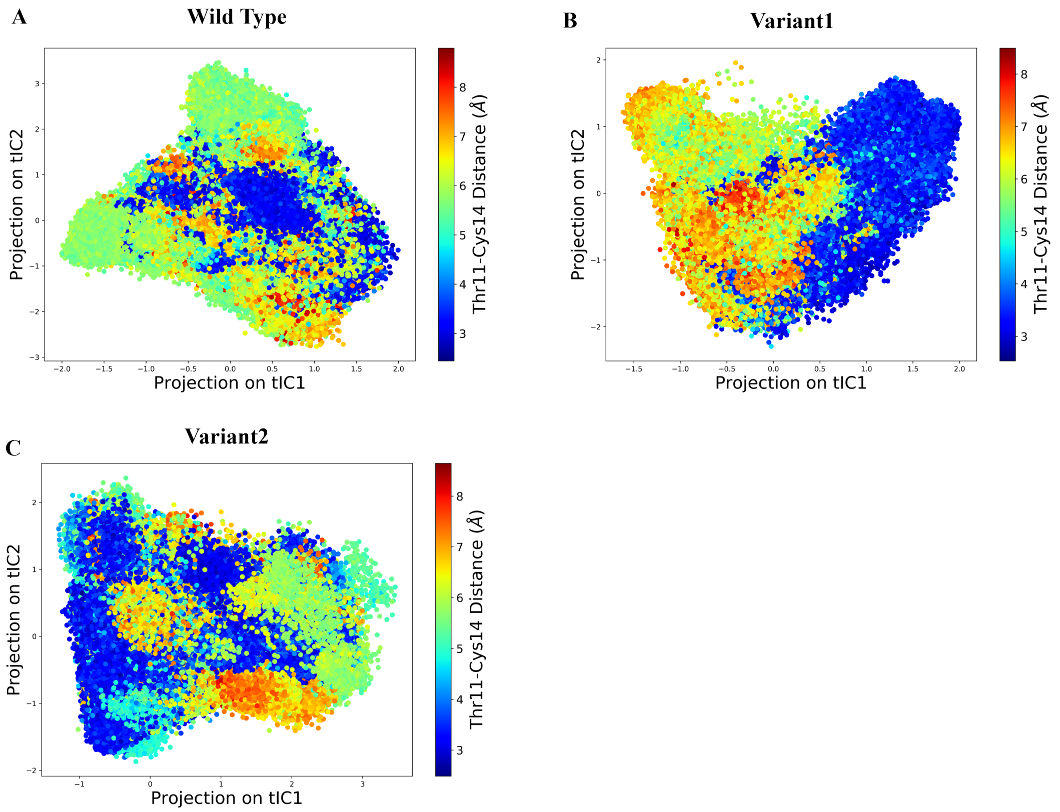


Figure S2: $(\mathrm{A}, \mathrm{B}, \mathrm{C})$ Variation of Thr11-Cys14 distance projected along tIC1 and tIC2 for ProcA3.3 core peptide variants. tIC1 and tIC2 are the first and second components of time-lagged independent component analysis (described in the Method section). They represent the two slowest components in the system. Thr11-Cys14 distance has a high correlation with tIC1, especially for the ProcA3.3 Variant1. Along with the changes in tIC1, Thr11-Cys14 distance shows a clear trend.

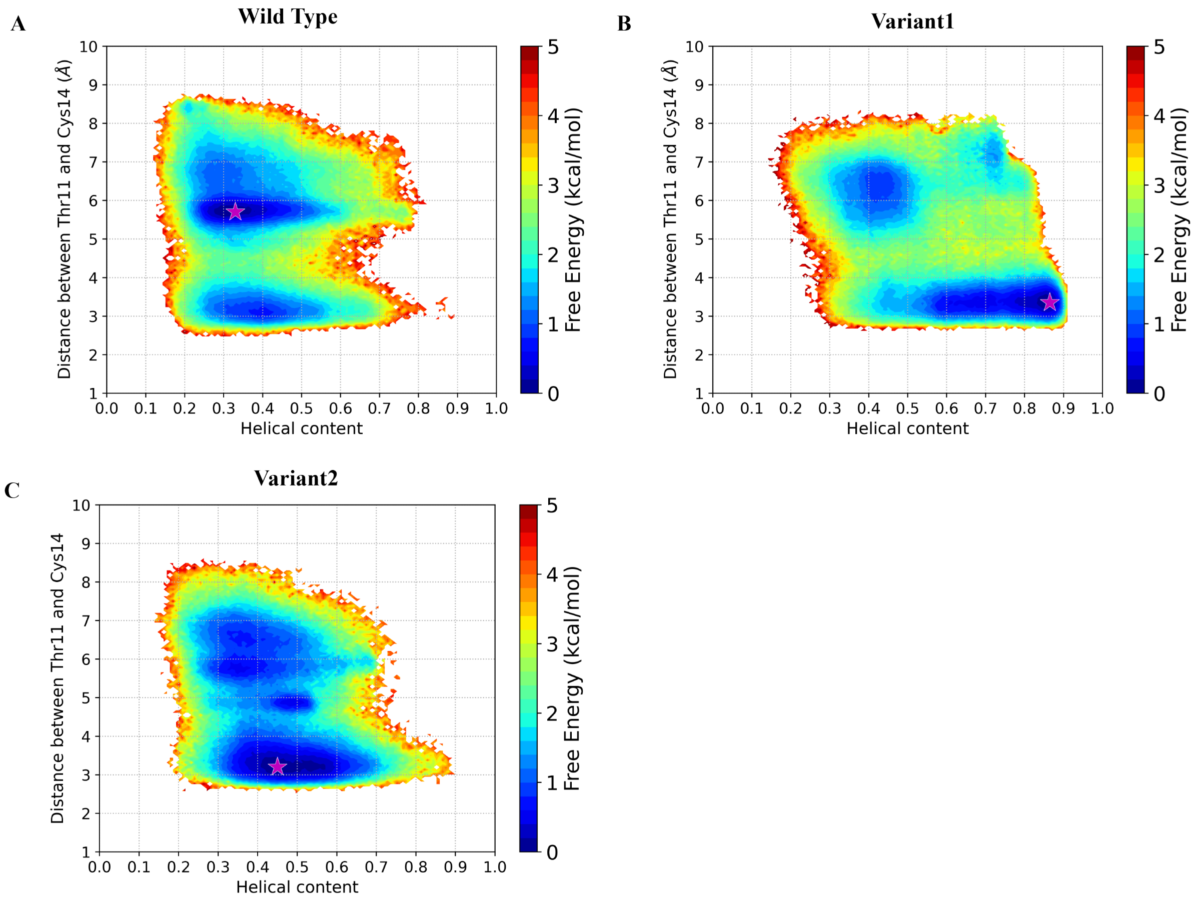


Figure S3: $(\mathrm{A}, \mathrm{B}, \mathrm{C})$ MSM (Markov state model) weighted free energy landscape projected along $\alpha$-helix content (located from Thr11 to Ala23) and Thr11-Cys14 distance (distance between any of their heavy atoms) for ProcA3.3 variants. Star region is the most populated state in the system.

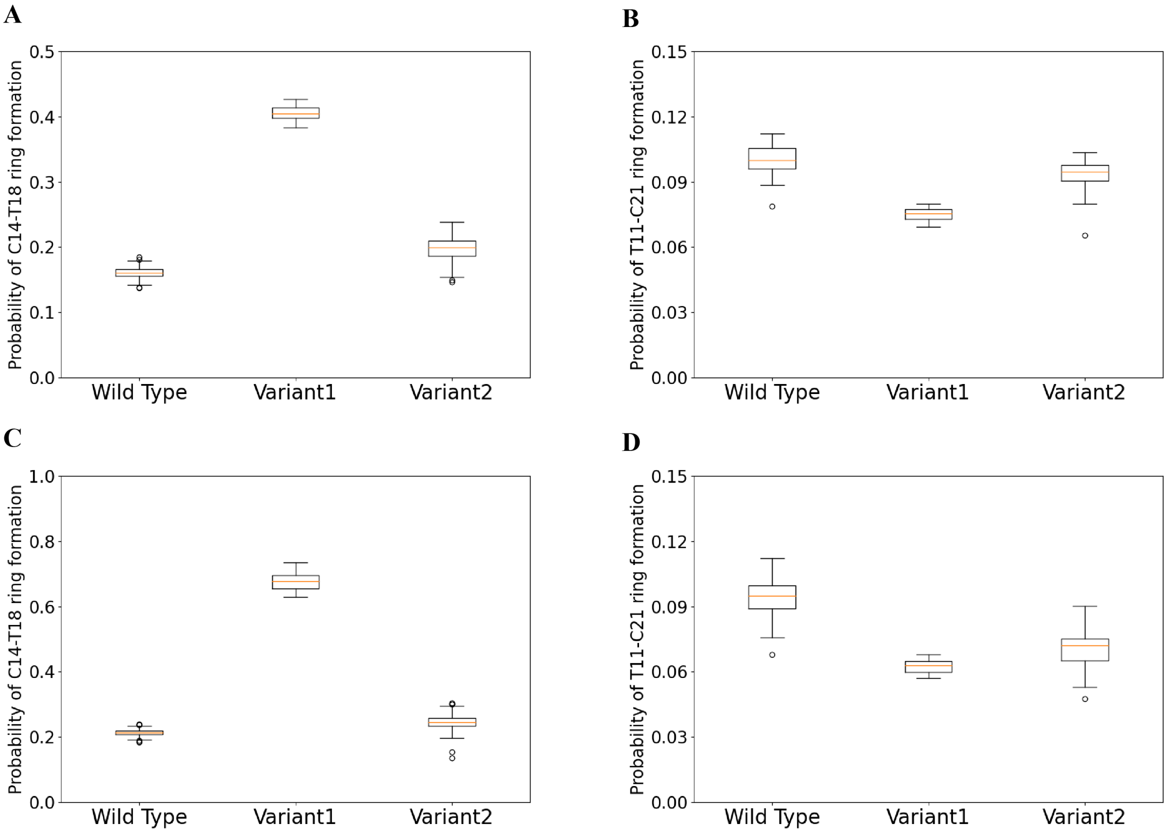


Figure S4: (A, B) Probability of formation of ring A (Cys14/Thr18) and B (Thr11/Cys21) for ProcA3.3 variants, with ring formation defined as the distance between the $\beta$-carbon of Thr and the sulfur of Cys being within 7.5 Å. (C, D) Probability of formation of ring A (Cys14/Thr18) and B (Thr11/Cys21) for ProcA3.3 variants, with ring formation defined as the distance between any of their heavy atoms being within 4.5 Å. Error bar was calculated by 200 bootstrap samples with 80% of total trajectories.

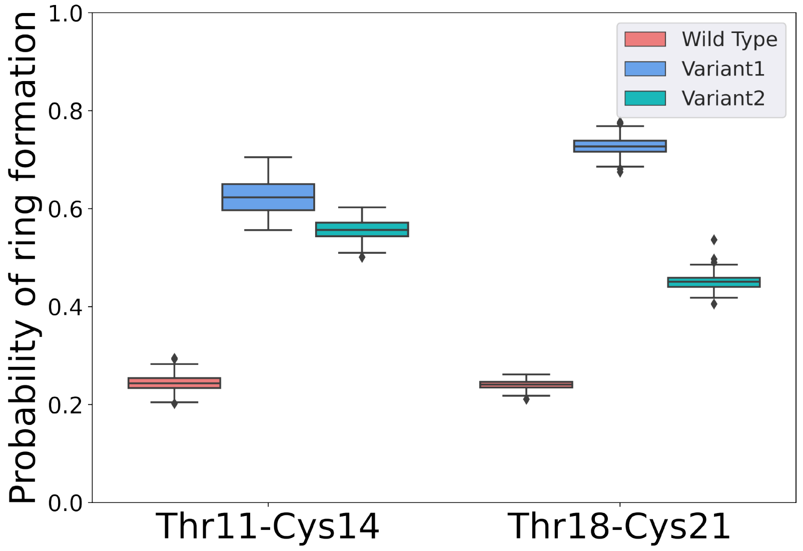


Figure S5: Probability of formation of ring $A^{'}$ (Thr11/Cys14) and $B^{'}$(Thr18/Cys21) for ProcA3.3 variants, with ring formation defined as the distance between any of their heavy atoms being within 4.5 Å. Error bar was calculated by 200 bootstrap samples with 80% of total trajectories.

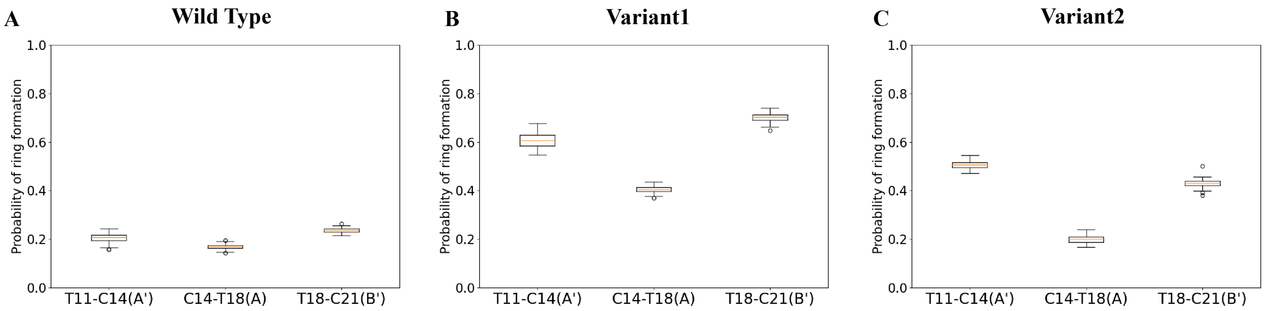


Figure S6: $(\mathrm{A}, \mathrm{B}, \mathrm{C})$ Probability of formation of ring $\mathrm{A}^{'}$ (Thr11/Cys14), ring A (Cys14/Thr18) and ring $\mathrm{B}^{'}$ (Thr18/Cys21) for ProcA3.3 variants. Error bar was calculated by 200 bootstrap samples with 80% of total trajectories.

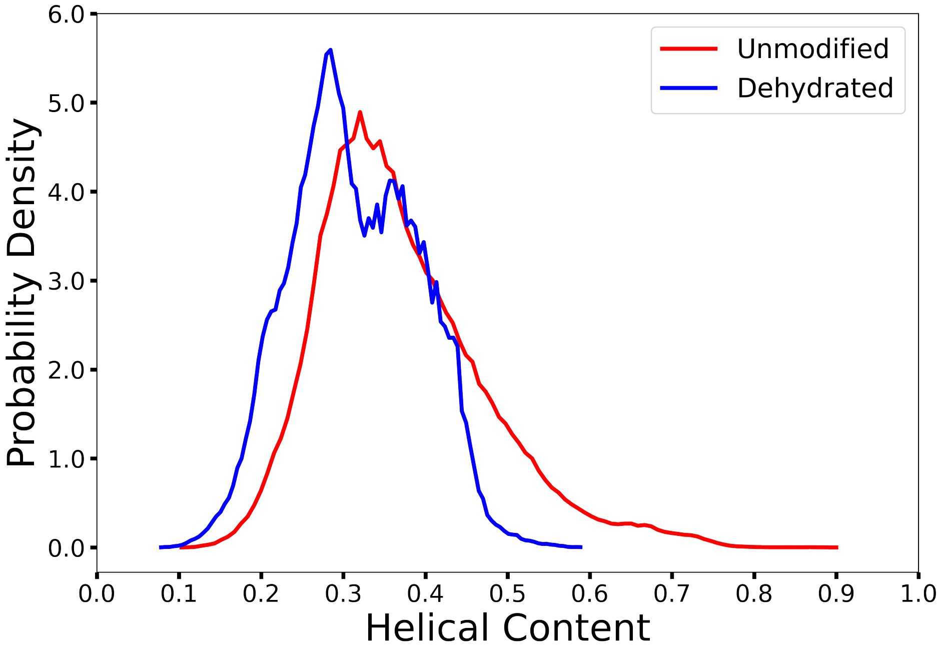


Figure S7: Comparison of the distribution of helical content for unmodified and fully dehydrated WT ProcA3.3 core peptide.

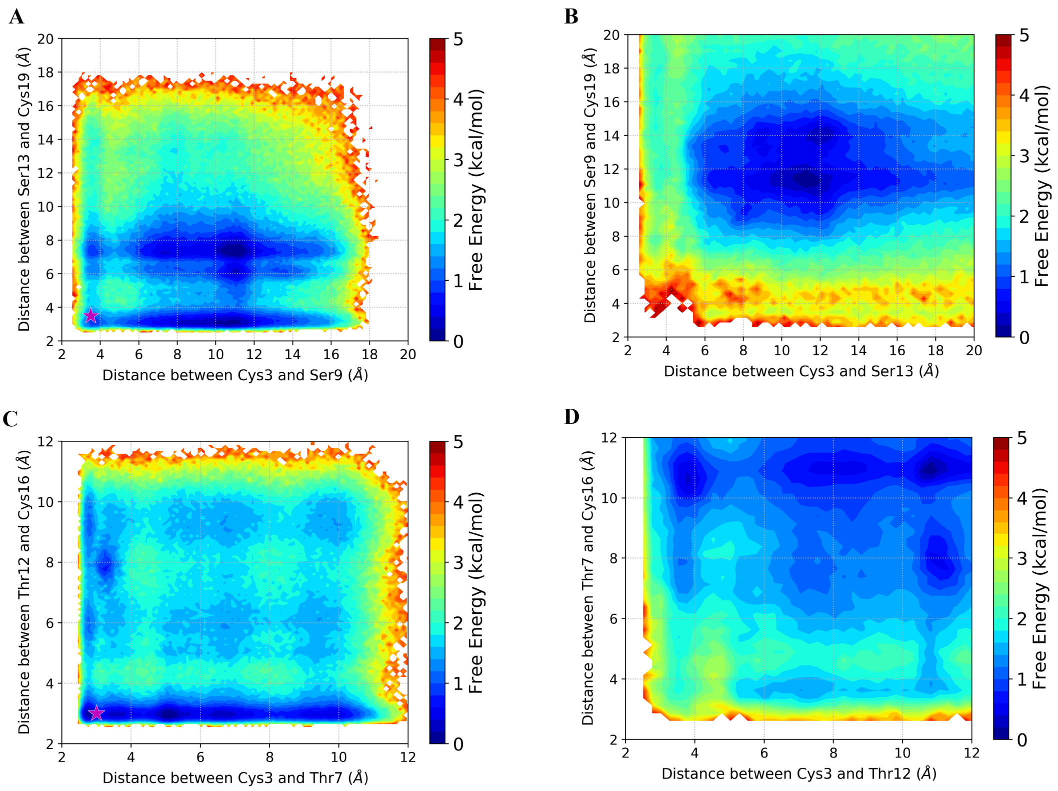


Figure S8: (A) MSM (Markov state model) weighted free energy landscape projected along the Cys3-Ser9 distance and the Ser13-Cys19 distance for ProcA2.8. (B) MSM weighted free energy landscape projected along the Cys3-Ser13 distance and the Ser9-Cys19 distance for ProcA2.8. (C) MSM weighted free energy landscape projected along the Cys3-Thr7 distance and the Thr12-Cys16 distance for ProcA1.1. (D) MSM weighted free energy landscape projected along the Cys3-Thr12 distance and the Thr7-Cys16 distance for ProcA1.1. Star region shows the state leading to rings A and B in the free energy landscape.

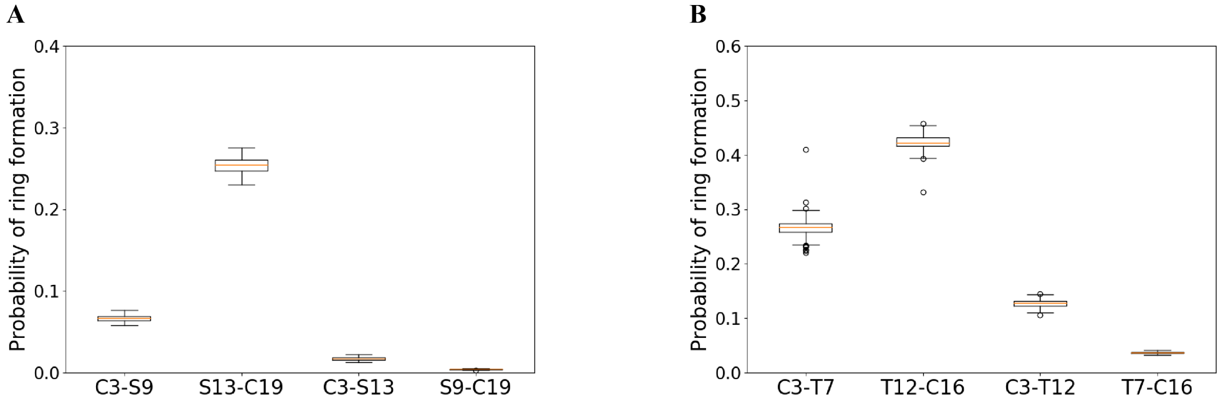


Figure S9: $(\mathrm{A}, \mathrm{B})$ Probability of formation of all possible rings for ProcA2.8 and ProcA1.1, with ring formation defined as the distance between any of their heavy atoms being within 4.5 Å. Error bar was calculated by 100 bootstrap samples with 80% of total trajectories.

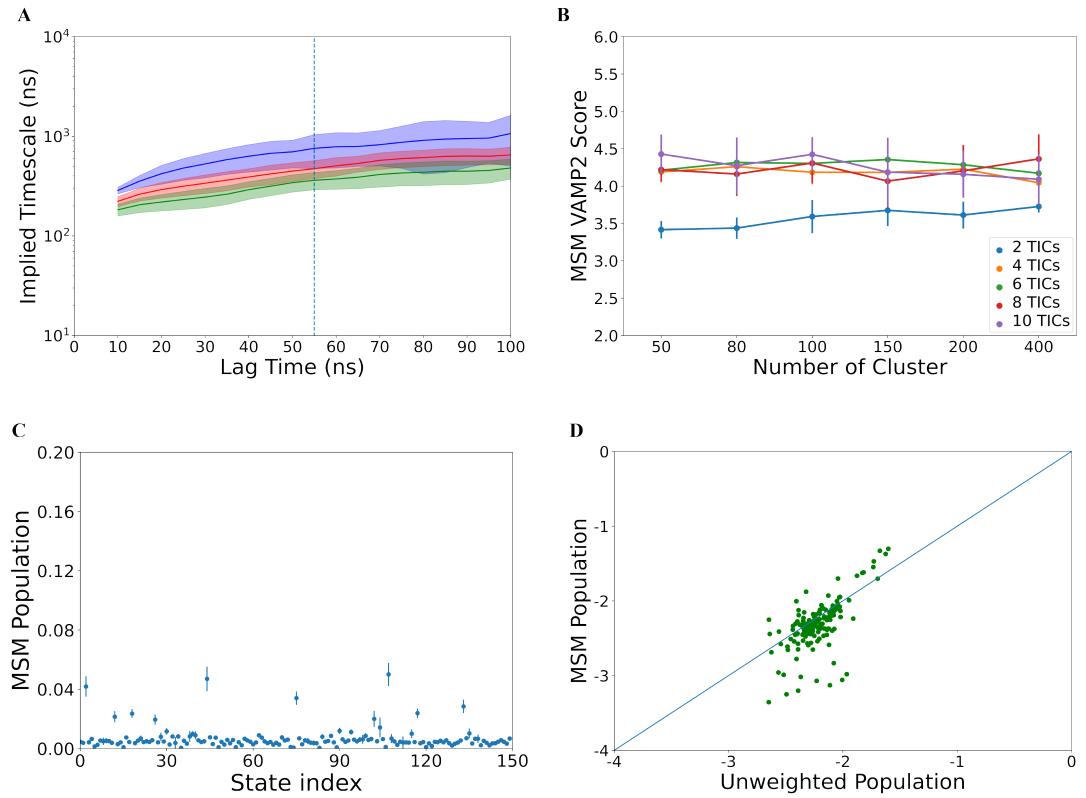


Figure S10: **Markov state model construction and analysis for ProcA3.3 WT.** (A) Implied timescale calculated from the 2nd (blue), 3rd (red) and 4th (green) highest MSM eigenvalues at different lag times, MSM lagtime of 55 ns was chosen. (B) VAMP-2 scores with different clusters and different number of tICs of MSM, 150 clusters and 6 tic dimensions were selected for MSM. (C) MSM population density of different MSM state index, error bar was calculated by 200 rounds bootstrapping with 80% of total trajectories. (D) MSM population vs Raw population in each state.

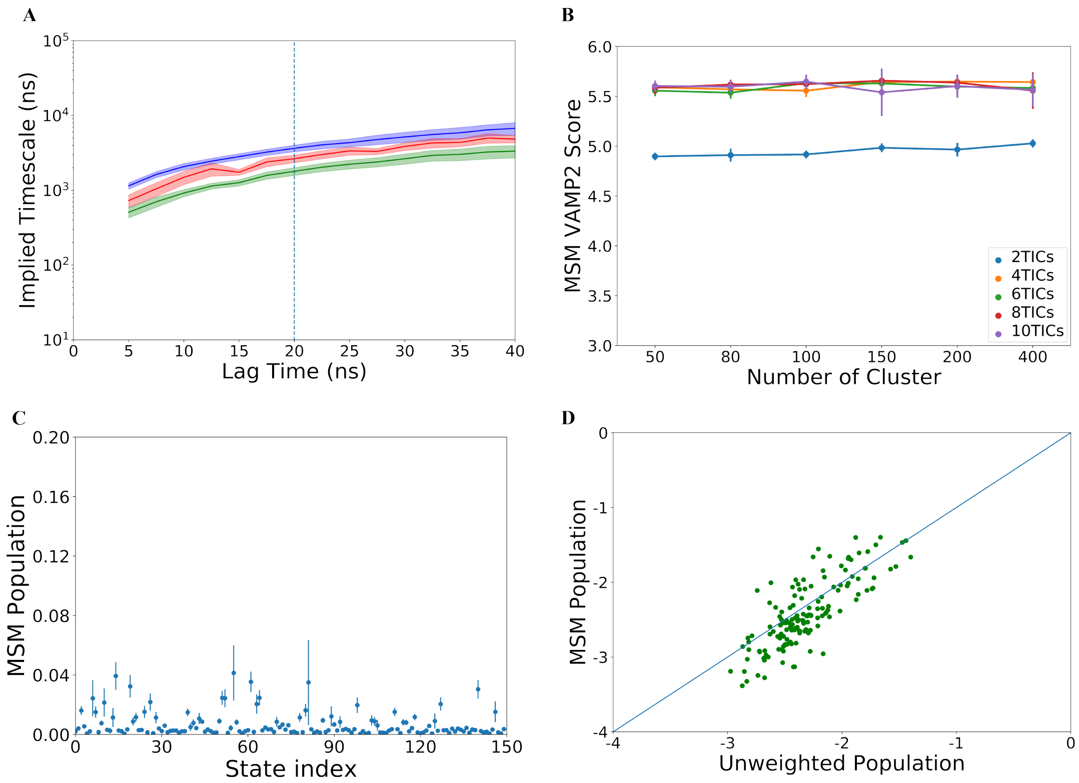


Figure S11: **Markov state model construction and analysis for ProcA3.3 Variant1.** (A) Implied timescale calculated from the 2nd (blue), 3rd (red) and 4th (green) highest MSM eigenvalues at different lag times, MSM lagtime of 20 ns was chosen. (B) VAMP-2 scores with different clusters and different number of tICs of MSM, 150 clusters and 8 tic dimensions were selected for MSM. (C) MSM population density of different MSM state index, error bar was calculated by 200 rounds bootstrapping with 80% of total trajectories. (D) MSM population vs Raw population in each state.

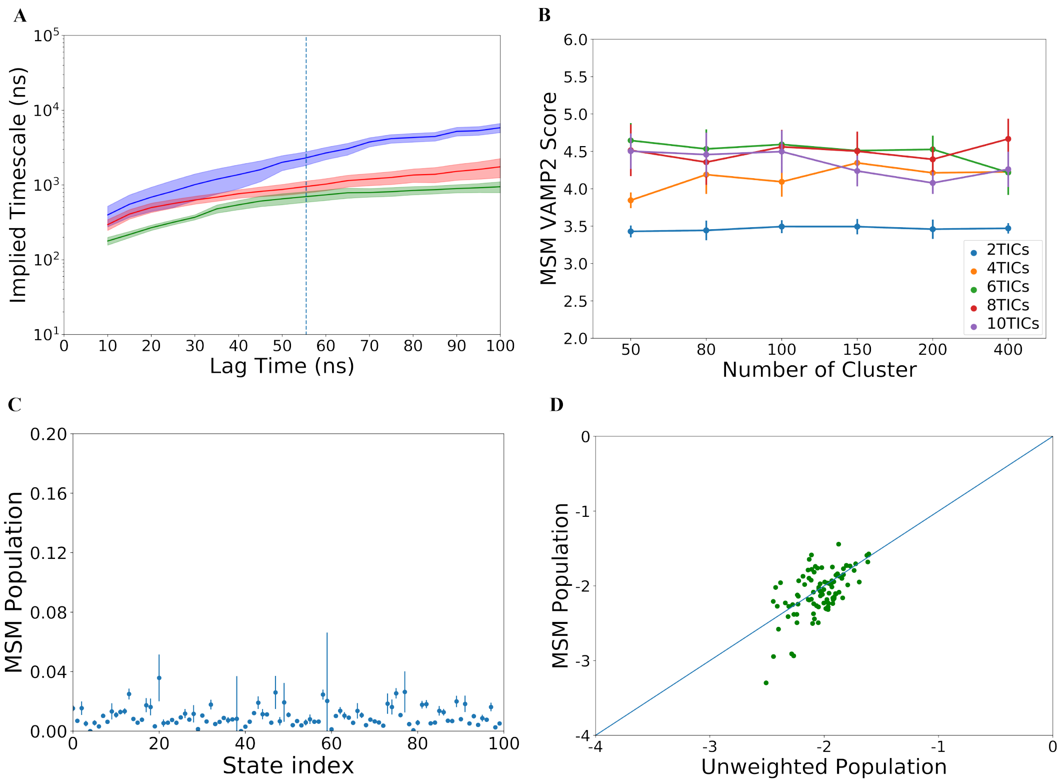


Figure S12: **Markov state model construction and analysis for ProcA3.3 Variant2.** (A) Implied timescale calculated from the 2nd (blue), 3rd (red) and 4th (green) highest MSM eigenvalues at different lag times, MSM lagtime of 55 ns was chosen. (B) VAMP-2 scores with different clusters and different number of tICs of MSM, 100 clusters and 6 tic dimensions were selected for MSM. (C) MSM population density of different MSM state index, error bar was calculated by 200 rounds bootstrapping with 80% of total trajectories. (D) MSM population vs Raw population in each state.

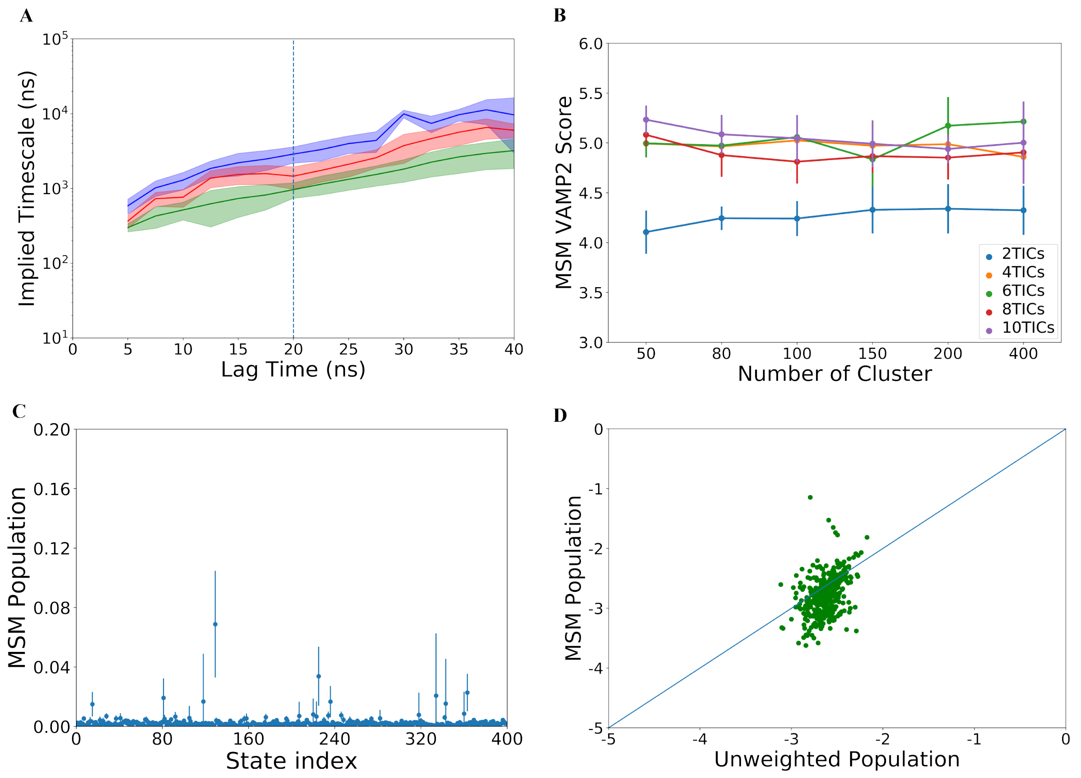


Figure S13: **Markov state model construction and analysis for dehydrated ProcA3.3 WT.** (A) Implied timescale calculated from the 2nd (blue), 3rd (red) and 4th (green) highest MSM eigenvalues at different lag times, MSM lagtime of 20 ns was chosen. (B) VAMP-2 scores with different clusters and different number of tICs of MSM, 400 clusters and 6 tic dimensions were selected for MSM. (C) MSM population density of different MSM state index, error bar was calculated by 200 rounds bootstrapping with 80% of total trajectories. (D) MSM population vs Raw population in each state.

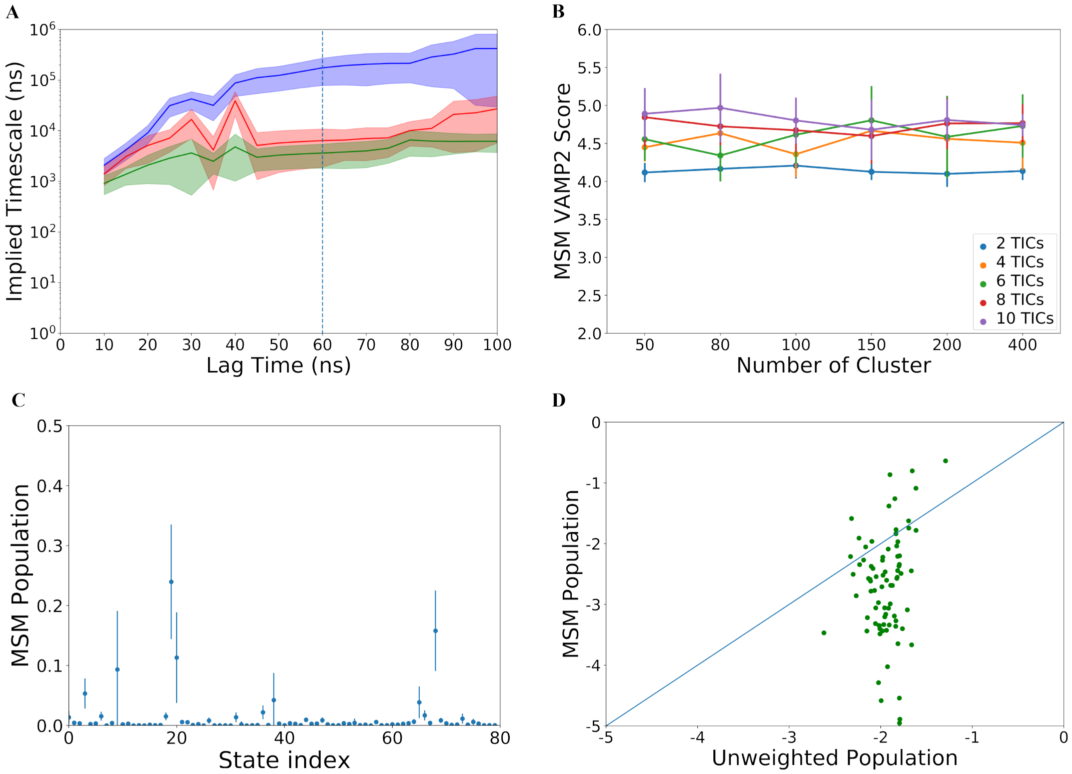


Figure S14: **Markov state model construction and analysis for dehydrated ProcA3.3 Variant1.** (A) Implied timescale calculated from the 2nd (blue), 3rd (red) and 4th (green) highest MSM eigenvalues at different lag times, MSM lagtime of 60 ns was chosen. (B) VAMP-2 scores with different clusters and different number of tICs of MSM, 80 clusters and 10 tic dimensions were selected for MSM. (C) MSM population density of different MSM state index, error bar was calculated by 200 rounds bootstrapping with 80% of total trajectories. (D) MSM population vs Raw population in each state.

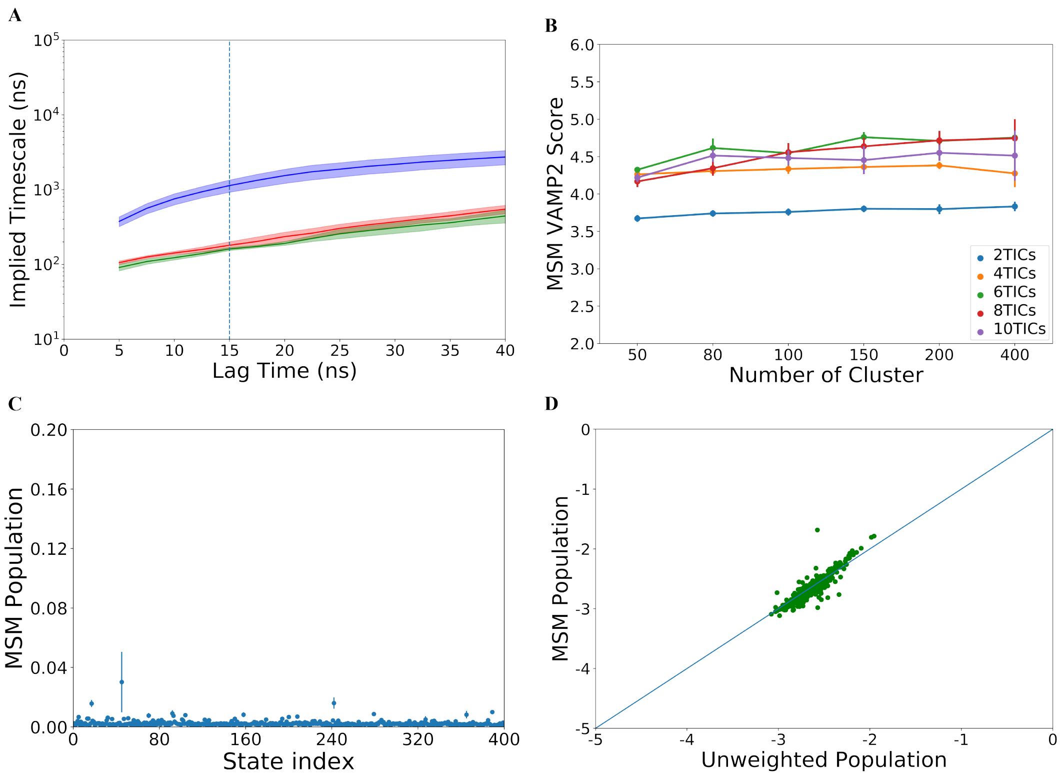


Figure S15: **Markov state model construction and analysis for ProcA1.1.** (A) Implied timescale calculated from the 2nd (blue), 3rd (red) and 4th (green) highest MSM eigenvalues at different lag times, MSM lagtime of 15 ns was chosen. (B) VAMP-2 scores with different clusters and different number of tICs of MSM, 400 clusters and 8 tic dimensions were selected for MSM. (C) MSM population density of different MSM state index, error bar was calculated by 200 rounds bootstrapping with 80% of total trajectories. (D) MSM population vs Raw population in each state.

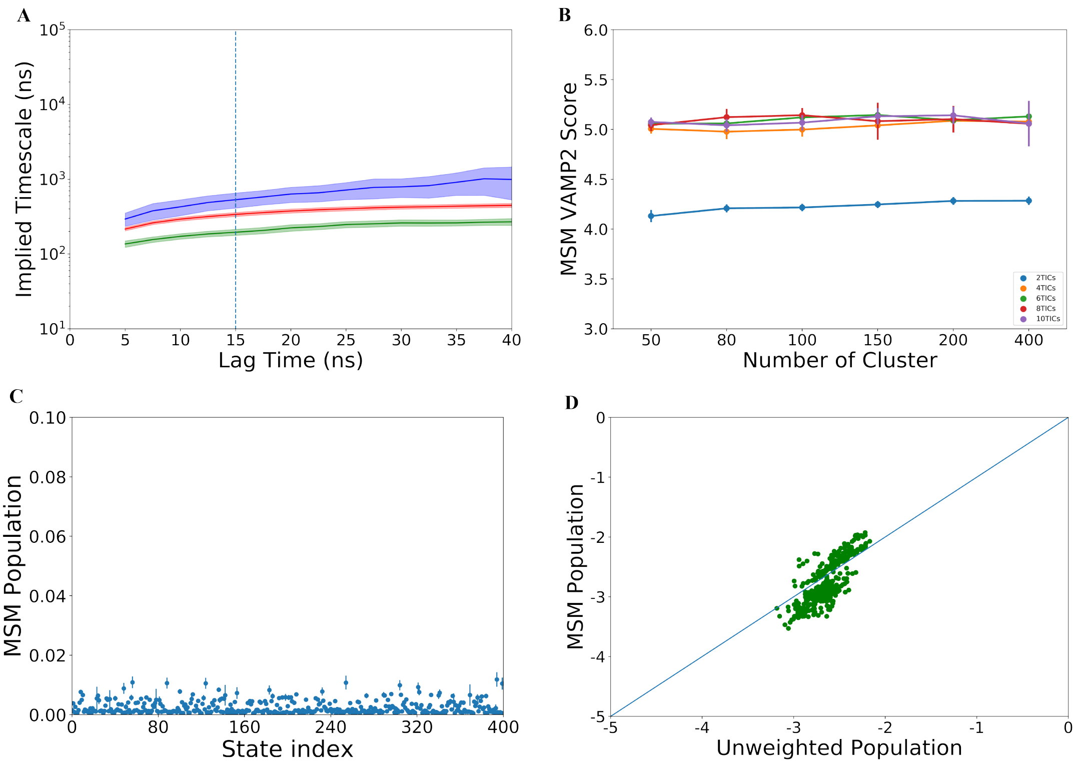


Figure S16: **Markov state model construction and analysis for ProcA2.8.** (A) Implied timescale calculated from the 2nd (blue), 3rd (red) and 4th (green) highest MSM eigenvalues at different lag times, MSM lagtime of 15 ns was chosen. (B) VAMP-2 scores with different clusters and different number of tICs of MSM, 400 clusters and 6 tic dimensions were selected for MSM. (C) MSM population density of different MSM state index, error bar was calculated by 200 rounds bootstrapping with 80% of total trajectories. (D) MSM population vs Raw population in each state.

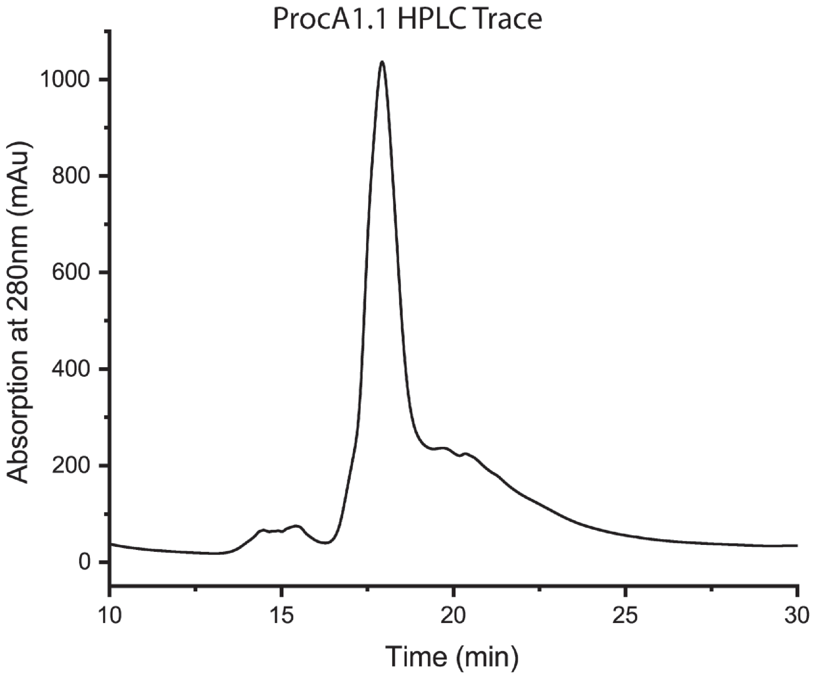


Figure S17: Chromatogram of ProcA1.1 purified by high-performance liquid chromatography. ProcA1.1 elutes with a retention time of 18 minutes.

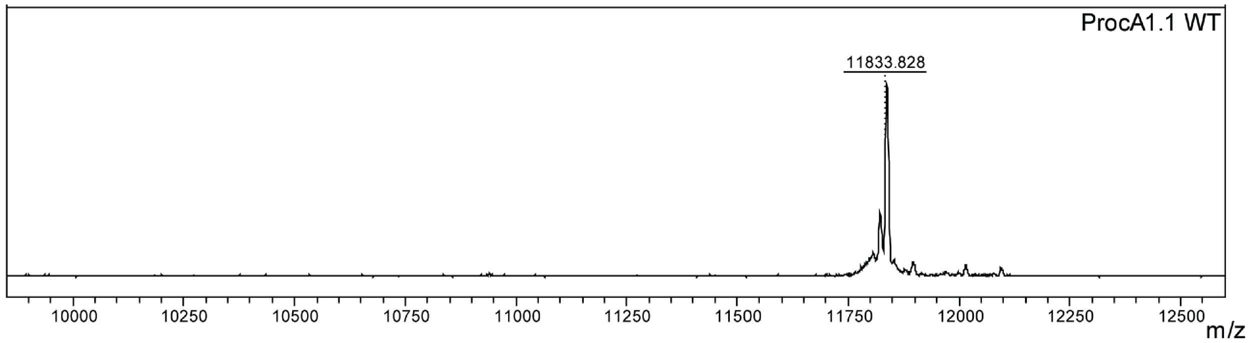


Figure S18: MALDI-TOF mass spectrum of HPLC purified unmodified ProcA1.1 (calculated m/z= 11833.832 ;observed= 11833.828).

Table S1: Molecular Dynamic Simulation Systems

| Peptide | Status | Sequence | Simulation Time |
|---|---|---|---|
| ProcA3.3 WildType | Unmodified | GDTGIQAVLHTAGCYGGTKMCRA | 40$\mu$s |
| ProcA3.3 WildType | Dehydrated | GD**Dhb**GIQAVLH**Dhb**AGCYGG**Dhb**KMCRA | 25$\mu$s |
| ProcA3.3 Variant1 | Unmodified | GDTGIQAVLHTLICFVITHLCRA | 40$\mu$s |
| ProcA3.3 Variant1 | Dehydrated | GD**Dhb**GIQAVLH**Dhb**LICFVI**Dhb**HLCRA | 25$\mu$s |
| ProcA3.3 Variant2 | Unmodified | GDTGIQAVLHTLFCYGGTFDCRA | 40$\mu$s |
| ProcA1.1 | Unmodified | FFCVQGTANRFTINVC | 25$\mu$s |
| ProcA2.8 | Unmodified | AACHNHAPSMPPSYWEGEC | 25$\mu$s |

Residues in red are residues varied in ProcA3.3 variants.
